# MRI Based Radiomics as an Imaging Biomarker for Locally Advanced Carcinoma Rectum: Predicting Tumor Response Following Neoadjuvant Chemoradiotherapy as an Organ Preservation Strategy

**DOI:** 10.1002/cnr2.70574

**Published:** 2026-05-19

**Authors:** Abhinav Puppalwar, Reena Engineer, Ashish Kumar Jha, Shivakumar Gudi, Rahul Krishnartry, Prashant Nayak, Jai Prakash Agarwal, Jayant S. Goda

**Affiliations:** ^1^ Department of Radiation Oncology Tata Memorial Centre Mumbai India; ^2^ Homi Bhaba National Institute Anushakti Nagar Mumbai India; ^3^ Department of Nuclear Medicine and Molecular Imaging Tata Memorial Centre Mumbai India

**Keywords:** machine learning classifiers, Neoadjuvant chemoradiotherapy, radiomic signatures, rectal cancer, tumour response

## Abstract

**Background:**

Patients with locally advanced rectal cancer show variable responses to neoadjuvant chemoradiotherapy (NACTRT), making early prediction of treatment response clinically important. Radiomics and machine learning may offer a potential non‐invasive imaging biomarker to identify patients who are more likely to respond to treatment.

**Aim:**

We investigated a machine learning‐based radiomic approach to predict tumor response (TR) to NACTRT in patients with adenocarcinoma of the rectum from baseline and post‐NACTRT magnetic resonance imaging (MRI).

**Methods:**

One hundred patients with locally advanced rectal cancer, who underwent pre‐ and post‐treatment MRI after NACTRT were included in the study after approval from the institutional ethics committee. One hundred twenty‐four radiomic features were extracted using the TexRad softwareTm, from pre‐ and post‐treatment T2W MRI, which included first‐order, GLCM, and shape features. Features were selected by the recursive feature elimination (RFE) algorithm. Five machine learning classifiers—support vector machine (SVM), random forest (RF), gradient boost (GB), Naive Bayes (NB) and AdaBoost (AB) classifiers were used to develop a prediction model for predicting TR.

**Results:**

Five radiomic features were selected using for TR using RFE using RF algorithm. The RF model with 10‐fold internal cross‐validation performed the best for predicting TR. The predictive performance of the RF model for TR was found to be AUC: 0.79 ± 0.15, accuracy: 0.72 ± 0.12, and precision: 0.77 ± 0.10 for the test cohort respectively.

**Conclusion:**

RF classifier based radiomic model provided a non‐invasive method to predict TR in patients of carcinoma rectum undergoing NACTRT treatment. The predictive potential of the radiomic features further requires validation in a larger cohort of patients in a multicentric study for clinical translation.

## 1 Introduction

Personalized therapy has fundamentally transformed the therapeutic approach to locally advanced rectal carcinoma (LARC), enabling treatment strategies tailored to individual tumor characteristics and patient profiles [1]. The standard of care for LARC in the contemporary era is a multimodal approach incorporating neoadjuvant chemoradiotherapy (NACTRT), followed by total mesorectal excision (TME) in nearly all patients, irrespective of tumor response (TR) [2, 3]. Following NACTRT, approximately 87.0% of the patients have a partial response (PR), leading to downstaging of disease, better resection margins following surgery, and a 3‐year PFS rate of 76.0% [4]. Given the favorable oncological outcomes with excellent long‐term clinical outcomes with NACTRT and the potential of surgical morbidity, organ‐preserving strategies have gained increasing attention. In carefully selected patients who achieve a radiological complete response (CR), a nonoperative “watch‐and‐wait” approach with structured surveillance may be considered as a viable management option [5, 6, 7] or a local excision could be considered in a select group of patients who have a PR [5, 8], while patients with poor response can be managed with additional systemic therapy, that is, total neoadjuvant therapy [9]. Accurate stratification of patients based on early treatment response has become increasingly important in the management of locally advanced rectal cancer (LARC). Such risk‐adapted approaches enable the consideration of less invasive, organ‐preserving strategies for good responders to NACTRT, while guiding the use of more intensive therapeutic interventions in patients with suboptimal response [10, 11]. Therefore, the identification of reliable prognostic biomarkers for assessing treatment response remains essential for optimizing and individualizing therapeutic decision‐making in LARC. Currently, qualitative assessment of anatomical and functional T2‐weighted and diffusion‐weighted magnetic resonance imaging (T2‐MRI and DW‐MRI) followed by digital rectal examination and endoscopy is considered the optimal diagnostic strategy for defining response to chemoradiation [12, 13]. MRI has inherent limitations in differentiating posttreatment fibrosis from intermixed viable tumor tissues. The interpretation of these changes on post‐therapy imaging is often subjective, resulting in variable diagnostic performance, with reported sensitivity ranging from 11% to 71% and specificity as low as 19% for identifying complete responders [14]. These limitations underscores the need for more accurate, reliable, and objective noninvasive approaches for assessing treatment response.

Interest in image biomarker development and validation in patients with cancer is gaining popularity among researchers [15, 16, 17]. Radiomic‐derived quantitative biomarkers have gained increasing attention as objective imaging‐based metrics, with potential utility as predictive indicators of treatment response and clinical outcomes [15, 16, 17, 18, 19]. When integrated with machine learning (ML)‐based predictive modeling, these approaches enable the derivation of novel imaging biomarkers for treatment response assessment, with significant potential to advance precision‐driven therapeutic strategies in oncology [20, 21, 22, 23, 24, 25]. Radiomics has already demonstrated considerable potential for the prediction of tumor aggressiveness [26, 27, 28, 29, 30, 31]. In rectal cancer, radiomic features extracted from whole‐tumor volume have been shown to outperform the combination of T2 and DWI in assessing CR [32]. Wen et al. developed a clinicoradiomic nomogram to predict pathological complete response (pCR) in LARC patients undergoing NACTRT. Combining radiomic features from MRI with clinical factors, their model showed strong predictive performance (AUC 0.86 in the training cohort and 0.83 in the validation cohort), potentially aiding personalized treatment strategies [33]. The study by Cantürk et al. explored using radiomics to predict how well patients with LARC respond to NACTRT. The study investigators analyzed MRI data within a cohort of 101 patients, with particular emphasis on quantitative assessment of tumor and mesorectal characteristics. The results showed that combining tumor and mesorectum radiomic features improved predictive accuracy, with an AUC of 0.837, outperforming models using tumor or mesorectum data alone [34]. Prior studies have generally been limited by the evaluation of a narrow range of ML classifiers, along with a lack of standardization in MRI‐based radiomic feature extraction. In addition, many have not incorporated rigorous validation frameworks, such as cross‐validation and external validation, thereby restricting the robustness and generalizability of their findings. Our study compares multiple algorithms using standardized TexRAD software for radiomic extraction and cross‐validation to address these gaps. In this study, we sought to explore the accuracy of radiomic features using various ML classifiers in predicting TR to NACTRT in patients with LARC. We aimed to demonstrate the effectiveness of these signatures using established performance metrics as a potentially useful imaging biomarker, representing an initial step toward clinical application in predicting TR in patients with LARC undergoing NACTRT.

## 2 Materials and Methods

The following inclusion and exclusion criteria were applied to select the patient on retrospective data collection.

### 2.1 Inclusion Criteria

- Patients with LARC who completed the treatment based on the institutional protocol.
- Patients for whom a baseline MRI scan was available.

### 2.2 Exclusion Criteria

- Patients for whom baseline MRI scans were not available.
- Patients in whom the intended treatment could not be completed or whose treatment protocol was modified. Patients diagnosed with a second primary cancer.

### 2.3 Study Population

**FIGURE 1:**
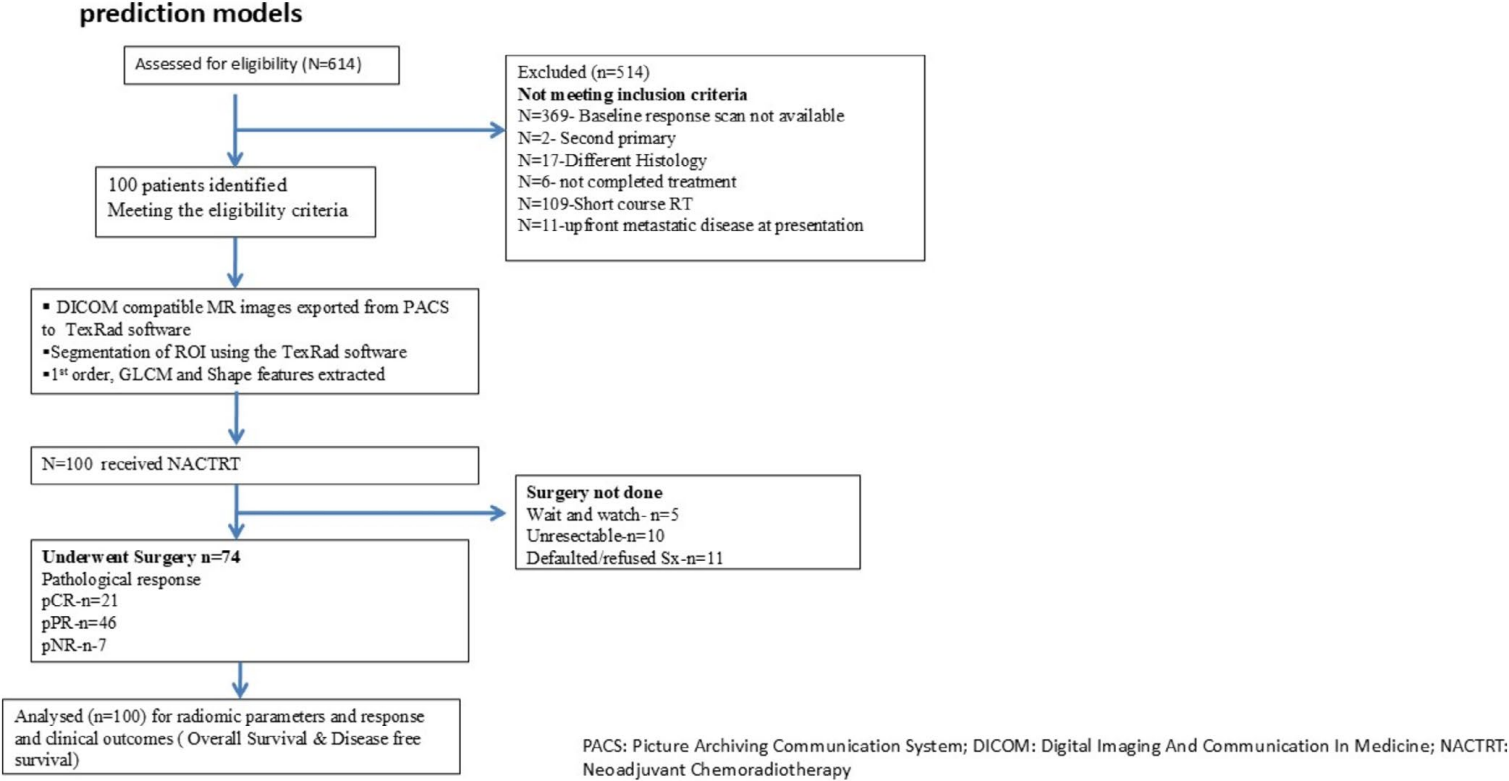
The consort flow diagram summarizes selection of rectal cancer patients for development of prediction models.

A total of 614 patients diagnosed with rectal carcinoma between 2015 and 2018 were screened for the study. One hundred patients diagnosed with LARC (Stage‐II/III) treated with NACTRT, and for whom baseline and posttreatment MRI data were retrievable, were included for analysis. Of the 514 excluded patients, 369 did not have a baseline MRI scan, 109 had undergone short‐course RT, 17 had alternative histology, 11 had metastatic disease, 6 did not complete the intended treatment, and 2 had a second primary cancer (Figure 1).

The NACTRT regimen consisted of capecitabine and oxaliplatin with image‐guided intensity‐modulated radiotherapy to a dose of 45 to 55 Gy administered in 25 to 33 fractions over 5–6 weeks.

All patients underwent an MRI scan to delineate disease extent and for treatment planning. T2‐weighted MRI sequences were utilized for radiomic analysis. The MRI DICOM images (Digital Imaging and Communications in Medicine) were obtained and transferred to the planning workstation, where the commercially available texture analysis research software TexRAD (Feedback Medical Ltd., Cambridge, UK) was used for tumor delineation and radiomic feature extraction.

### 2.4 Ethics Statement

Ethical approval was obtained from the Institutional Ethics Committee. All experimental methods were conducted according to the ethical guidelines for the use of human subjects. Being a retrospective study, a waiver of consent was granted by the ethics committee.

### 2.5 Outcome Measures and Classification of Clinical Data

One hundred patients with rectal cancer who underwent treatment in our hospital were included in this study. Patients' demographic and treatment data are shown in Table 1. Clinical data extracted from the hospital information system were cleaned and converted into a format suitable for training and validating the ML classifiers. Treatment response was selected as the clinical endpoint to develop prediction models. Clinical outcomes of the patients were binarized into CR = 1 and no/partial response (PR) = 0. The CR was defined primarily as clinical complete response (cCR), assessed using posttreatment MRI, endoscopy, and digital rectal examination findings. For patients who underwent surgery, pCR was used based on the histopathological report.

**TABLE 1:** Baseline patients profile and treatment profile.

Baseline characteristics (*N* = 100)	
Age: Median 50 years (range from 25 to 75 years)	
< 50 years	51
> 50 years	49
Gender	
Male	76
Female	24
Baseline MRI features	
Tumor size	6.2 cm (mean)
Clinical stage (AJCC 8th edition)	
II	6
III	94
Treatment details	
Surgery	
Yes	74
No	26
Wait and watch	5
Unresectable tumor	10
Defaulted/refused surgery	11
Radiotherapy	
Yes	100
No	0
Neoadjuvant radio‐chemotherapy	
Yes	100
No	0
Response to neadjuvant chemo radiotherapy (NACTRT)	
Complete response	33
Partial/no response	66

*Note:* Radiotherapy boost was given in 21 pts; concurrent chemotherapy with capecitabine was given in 95 pts and 5 fluorouracil was given in 5 pts.

Abbreviations: AJCC, American Joint Committee on Cancer; MRI, magnetic resonance imaging.

### 2.6 Radiomic Pipeline

**FIGURE 2:**
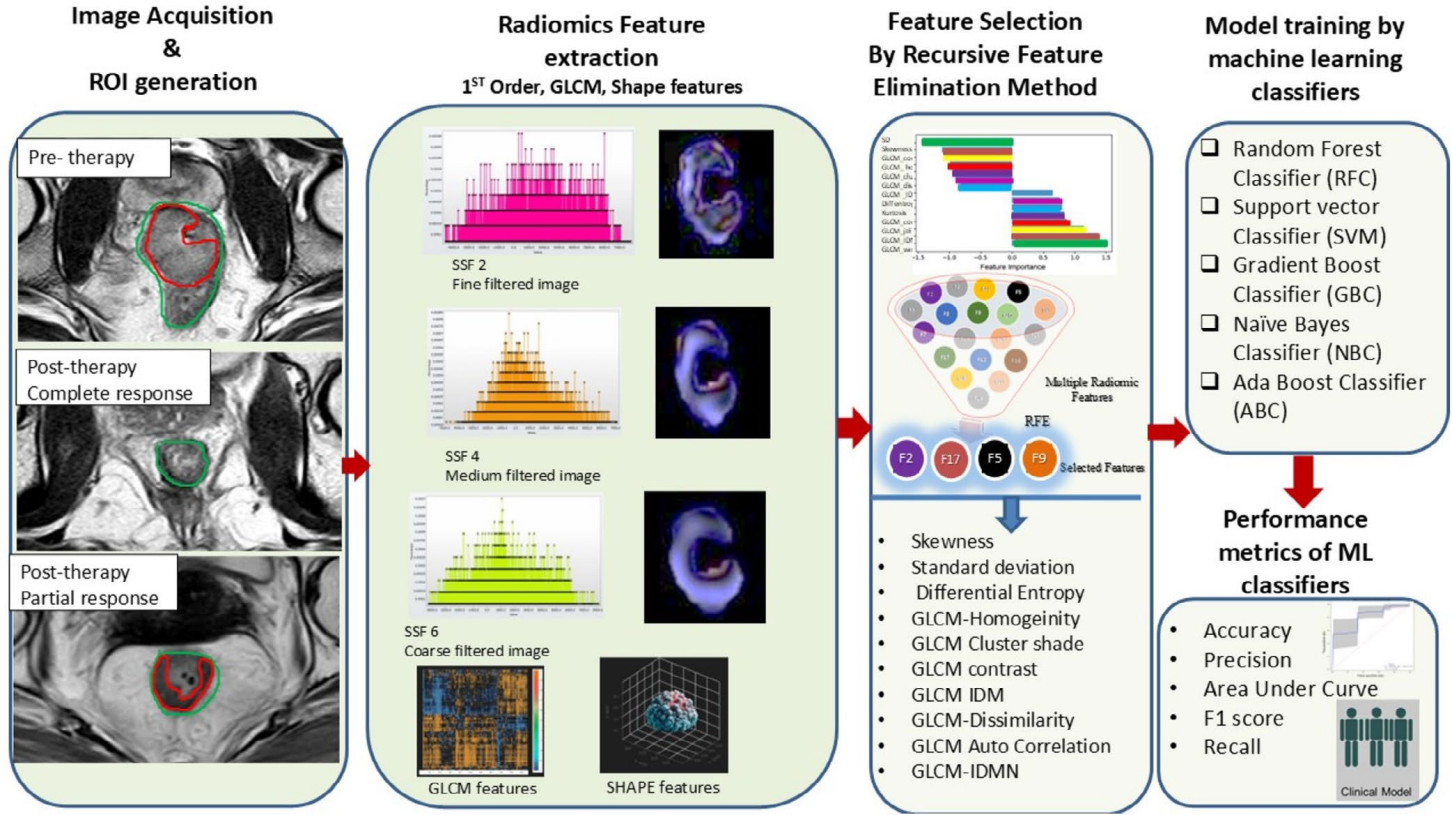
Workflow of image acquisition, segmentation, radiomics feature extraction, feature reduction, and building the model by machine learning classifiers (radiomics pipeline).

A schematic representation of the radiomic workflow is illustrated in Figure 2, outlining the sequential steps involved in image acquisition, segmentation, feature extraction, feature selection, and subsequent model development and validation.

### 2.7 Image Acquisition Protocol

MRI examinations were performed at our institution using a GE Signa 1.5 Tesla scanner, following a standardized, predefined protocol optimized for rectal tumor imaging. The acquired images were stored in the Picture Archiving and Communication System (PACS) and subsequently exported for radiomic (texture) analysis using TexRAD software.

### 2.8 MRI Pre‐Processing, Segmentation (ROI Generation)

Pelvic Images were acquired on a 1.5 Tesla General Electric (GE Signa) machine. The acquisition details are explicitly described in Table S1. The imaging protocol introduced some heterogeneity, reflecting differences in MRI scanners and sequence parameters inherent to retrospective data collection.

To address this prior to segmentation and ROI delineation, images were preprocessed using Laplacian of Gaussian (LoG) bandpass filters, which reduced background noise via Gaussian smoothing and enhanced tumor edges through the Laplacian component. This allowed for the extraction of specific structures corresponding to the tumor in the image. Spatial scale filters (SSF) used filtration values of 0, 2, 3, 4, 5, and 6 mm in width (radius), representing the increasingly coarser level of texture scales for first‐order statistics [35]. Applying a filtration algorithm prior to feature extraction mitigates the impact of variations in imaging acquisition protocols and enhances the reliability of feature selection by eliminating features influenced by MRI noise and differences in imaging parameters. The ROI was drawn individually after adjusting the window level for best visualization; the tumor boundaries of the visible primary tumor were contoured on consecutive slices encompassing the entire craniocaudal extent while excluding the lumen and its contents. The ROIs were independently verified by a radiation oncologist with 20 years of experience in treating gastrointestinal malignancies and a gastrointestinal radiologist with more than 10 years of experience. Discrepancies in image interpretation between the radiation oncologists and radiologists were resolved through collaborative review and consensus agreement.

### 2.9 MRI Radiomic Analysis

The radiomic features were extracted from the segmented images using proprietary texture analysis research software (TexRAD Research Version 3.10, TexRAD Ltd., Cambridge, UK) [35]. TexRAD was chosen for its robust filtration‐histogram algorithm, validated in oncologic radiomic studies, which ensures standardized feature extraction. Unlike some open‐source alternatives like pyradiomics, TexRAD's approach has shown strong reproducibility across multiple studies. The variables consisted of 1st order texture features and shape (topographic) features. Each data point had 124 variables (features) extracted from the segmented images of the T2‐weighted sequences of both preoperative and postoperative MRI images. Thirty‐six first‐order features were extracted using various spatially scaled filters (0, 2, 3, 4, and 6), and 20 GLCM and 6 shape (topographic) features were extracted without applying filters. These radiomic features were then selected using the random forest (RF) elimination method and then modeled for various endpoints of interest. The details of all the radiomic features are provided in Table S2.

### 2.10 Data Normalization

Data normalization is a necessary step as it gives equal weightage to each variable, ensuring no single variable steers the model performance in a direction because they are big numbers. We used the Min–Max normalization (rescaling) technique to normalize the entire dataset.

### 2.11 Feature Reduction (Selection)

Feature selection is a critical preprocessing step in which redundant and non‐informative variables are eliminated, thereby reducing dimensionality while retaining the most relevant information from the original dataset. When performed before model training, this process helps, mitigate overfitting, reduces noise, and enables the identification of key features that are most strongly associated with the outcome of interest. Additionally, it enhances computational efficiency and contributes to improved model stability and overall predictive performance. Recursive feature elimination (RFE) with a RF algorithm was used on a pilot dataset of 30 patients and selected five optimal features from both preoperative and posttreatment MRI for classifying TR in an independent dataset. The RFE method removes the weakest feature (or features) until the specified number of features is reached. It identifies variables in multivariate analysis that are significant, independent predictors of the outcome of interest (i.e., class difference). The selected optimal features for the clinical endpoint of TR are shown in the Table 2.

### 2.12 Testing and Training of Models

**FIGURE 3:**
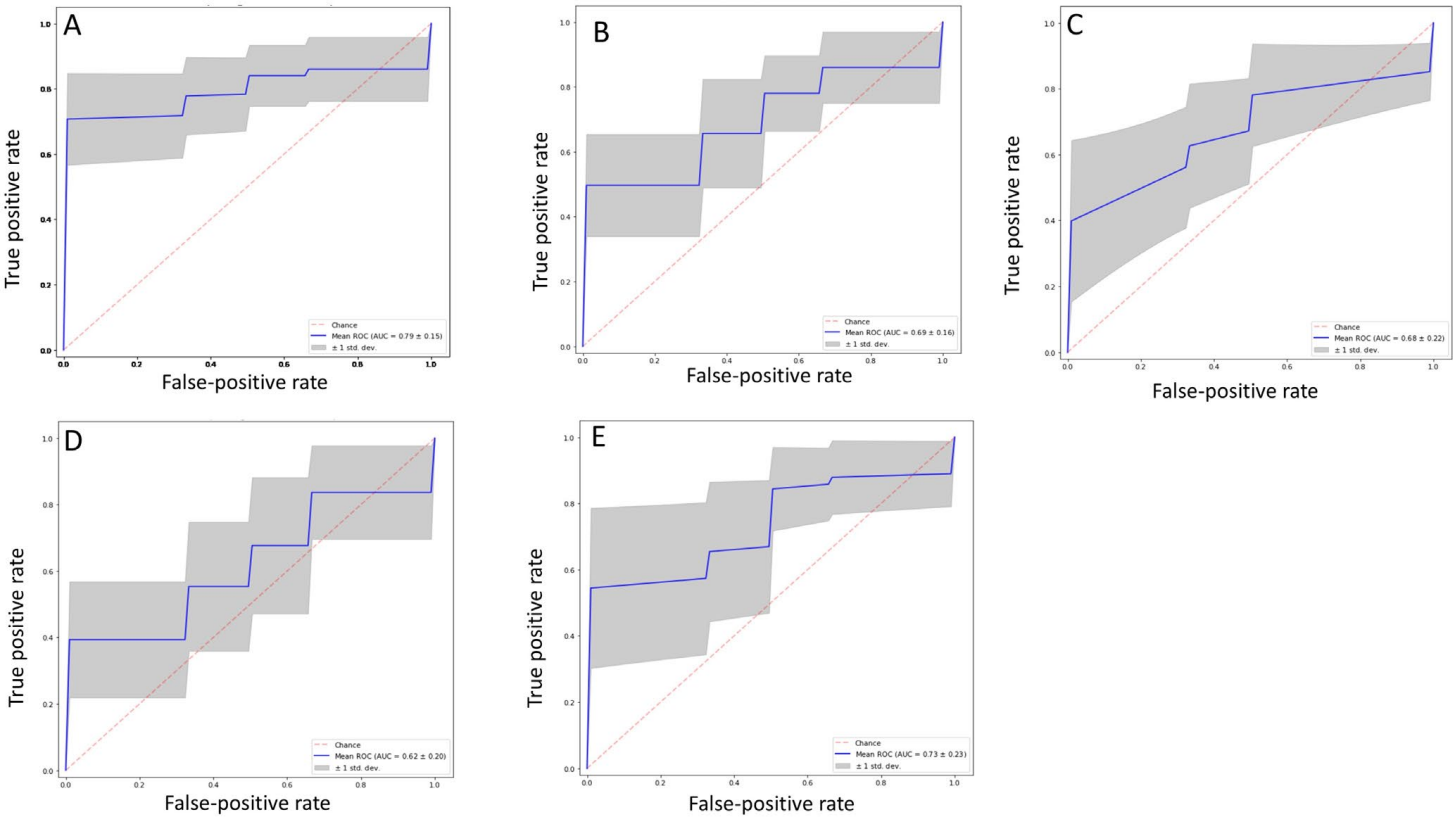
(A–E) Area under the curve (AUC) calculated from various machine learning algorithms for differentiating complete responders from non/partial responders.

A 10‐fold internal cross‐validation was performed in a dataset independent of the feature selection study on the dataset of 100 patients. The training data were split into 10 subsets, with one reserved for testing and the model trained on the remaining 9 subsets. This was then iterated 10 times. This data was used to train five classification models: random forest classifier (RFC), support vector machine classifier (SVM), gradient boosting classifier (GBC), Naive Bayes classifier (NBC), and AdaBoost classifier (ABC). The area under the receiver operating characteristic curve (AUC) was also calculated, and the curve was plotted for all models (Figure 3). The most important features were identified using the RFC, which is an ensemble ML algorithm comprising multiple decision trees that uses bootstrap aggregation.

SVMs are classification models that identify an optimal hyperplane to separate different classes. This boundary is defined by the closest data points from each class, known as support vectors, enabling effective performance in high‐dimensional feature spaces.

GBC is an ensemble learning method that builds a strong predictive model by sequentially combining multiple weak learners, usually decision trees. Each new model is trained to reduce the errors of the previous ones by optimizing a loss function, such as log loss, thereby progressively improving prediction accuracy.

NBCs are probabilistic models that handle uncertainty by using probabilities to describe how different variables relate to each other. In this framework, each feature is treated as a random variable, and the model predicts the most likely class by combining the influence of all these features, while assuming they are independent of one another.

Adaptive Boosting (AdaBoost) is an ensemble learning method that improves classification performance by combining multiple weak learners. It operates iteratively by adjusting the weights of training samples, increasing the emphasis on previously misclassified observations. Each subsequent model is trained to correct the errors of its predecessors, and the process continues until the specified number of estimators is reached or the model achieves satisfactory convergence.

Model validation was performed using cross‐validation, a resampling technique that assesses model generalizability by evaluating its performance on unseen data. We applied stratified 10‐fold cross‐validation and used a resampling method that uses different portions of the data to test and train a model on various iterations. Various prediction algorithms (RFC, SVM, GBC, NBC, ABC) were used to predict response using 10‐fold validation and studied compared with the ROC curve and AUC. The patient demographics were done using SPSS v. 21.0. (IBM SPSS Statistics for Windows, v. 21.0, NY). Descriptive statistics were expressed as numbers and percentages for categorical variables and mean ± standard deviation or median (interquartile range) for continuous variables.

### 2.13 The Prediction Model Assessment

For all the statistical tests, different packages of Python 3.9.0 software were used. Descriptive statistical tests were performed to understand the distribution of patients in various categories. The performance of prediction models was assessed using standard performance assessment matrices, that is, accuracy, precision, recall, *F*1‐score, and AUC. Details about prediction matrices are shown in Table S3.

## 3 Results

### 3.1 Baseline Demographics, Tumor, and Treatment Characteristics of the Study Cohort

The median age of the cohort was 50 years, with a higher male preponderance. One hundred patients were identified for the study who underwent the intended therapy (NACTRT). Following NACTRT, 74 patients underwent surgery while, 5 patients had a complete clinical‐radiological response and were put on a wait and watch protocol, 10 patients had unresectable disease while 11 had refused to undergo surgery. All the tumors were locally advanced with a mean size of 6.22 cm. The majority of patients (46%) had a histological diagnosis of moderately differentiated adenocarcinoma, while T3 disease was observed in 73% of patients and stage IIIB disease in 73% of patients, respectively. The demographic and treatment details are shown in Table 1.

### 3.2 Selected Features for the Clinical Endpoints Used in Model Development

The RFE selected the optimal and stable radiomic features for each of the clinical endpoints chosen. The most stable radiomic features selected from the RFE method were then used for model building using various ML algorithms with a 10‐fold stratified cross‐validation strategy. The top five features for the endpoint of interest (Tumor Response) to NACTRT (i.e., class difference of CR vs. PR) were standard deviation from fine filtered (SSF2) images on baseline MRI, standard deviation from medium filtered images (SSF3) on baseline MRI, skewness from fine filtered (SSF2) images on baseline MRI, GLCM‐IDMN (grey level co‐occurrence matrix‐inverse difference moment normalized) on Post‐NACTRT MRI and differential entropy on post‐NACTRT MRI (Table 2).

**TABLE 2:** Radiomic features selected for model development for clinical endpoints.

Clinical endpoint criteria	Feature selection method	Selected optimal radiomic features from baseline and postoperative MRI
Tumor response to NACTRT	Recursive feature elimination using random forest algorithm	Standard deviation (SSF3)—Baseline MRI Standard deviation (SSF2)—Baseline MRI Skewness (SSF2)—Baseline MRI GLCM‐IDMN—Post‐NACTRT MRI Differential entropy—NACTRT MRI

Abbreviations: GLCM, grey level co‐occurrence matrix; IDMN, inverse difference moment normalized; NACTRT, neoadjuvant chemoradiotherapy; SD, standard deviation; SSF, spatial scaled filter.

### 3.3 Test Performance Measures Using Various ML Classifiers

The performance of the five classifiers using a 10‐fold cross‐validation is shown in Table 3. Of the five classifier models, the RF model was found to be the most stable and performed better than the other four classifier for the endpoint of TR. For the prediction of TR to NACTRT, the RFC achieved a predictive performance (AUC: 0.79 ± 0.15, accuracy: 0.72 ± 0.12, *F*1‐score: 0.8 1 ± 0.07, recall: 0.87 ± 0.11, and precision: 0.77 ± 0.10) for the test cohort. It achieved the highest accuracy (0.72 ± 0.12), outperforming SVM (0.68 ± 0.16), GBC (0.67 ± 0.13), NBC (0.67 ± 0.06), and ABC classifiers (0.71 ± 0.12). Notably, RFC balances precision (0.77 ± 0.10) and recall (0.87 ± 0.11), resulting in the highest *F*1‐score (0.81 ± 0.07). Additionally, RFC's AUC (0.79 ± 0.15) surpassed other models, indicating strong discriminatory ability. The RFC demonstrated superior performance across all evaluated diagnostic metrics, indicating a strong overall discriminative capability among the models compared. Its consistently high values for sensitivity, specificity, accuracy, and AUC suggest robust classification performance and balanced error control. However, the relatively wide confidence intervals observed for both AUC and accuracy likely reflect statistical uncertainty attributable to the limited sample size. In the context of a 10‐fold cross‐validation framework, smaller datasets result in fewer observations within each fold, increasing variability in performance estimates across iterations. Consequently, this sampling variability can inflate the width of confidence intervals, even when the mean performance remains high. Given the small sample size, only 10 patients were allocated to the validation subset in each cross‐validation fold. Such a limited validation sample size reduces the precision of performance estimates and increases the variation in the performance metrics such as accuracy and AUC across the folds. We agree from a statistical perspective, small test sets yield fewer stable estimators of model performance, thereby contributing to wider confidence intervals and greater susceptibility to sampling variability.

**TABLE 3:** Prediction model performance from selected radiomic features for classifying tumour response (complete response vs. no/partial response).

Machine learning algorithm	Cross‐validation	Accuracy	Classification report			AUC
			Precision	Recall (sensitivity)	*F*1‐score	
Random forest classifier (RFC)	10‐folds	0.72 ± 0.12	0.77 ± 0.10	0.87 ± 0.11	0.81 ± 0.07	0.79 ± 0.15
Support vector machine classifier (SVM)	10‐folds	0.68 ± 0.16	0.73 ± 0.11	0.87 ± 0.14	0.79 ± 0.11	0.69 ± 0.16
Gradient boosting classifier (GBC)	10‐folds	0.67 ± 0.13	0.75 ± 0.08	0.77 ± 0.16	0.75 ± 0.11	0.68 ± 0.21
Naive Bayes classifier (NBC)	10‐folds	0.67 ± 0.06	0.61 ± 0.06	0.99 ± 0.04	0.80 ± 0.04	0.62 ± 0.20
AdaBoost classifier (ABC)	10‐folds	0.71 ± 0.12	0.79 ± 0.12	0.84 ± 0.14	0.80 ± 0.08	0.73 ± 0.23

*Note:* Each performance value was calculated by averaging the results of the 10‐fold cross‐validation; 1: complete response, 0: partial or no response.

Abbreviation: AUC, area under curve.

The other four classifiers, however, did not perform as well as the RFC with an AUC in the range of 0.69–0.73 and accuracy in the range of 0.68–0.71. Table 3 shows the prediction model performance of the ML classifiers for predicting complete responders versus partial responders following therapy with NACTRT using different performance metrics. Figure 3A–E shows the ROC curves for the five classifiers in the test cohort.

## 4 Discussion

Personalized treatment for rectal cancer relies on clinical and imaging markers to predict therapeutic outcomes. Among these, response to NACTRT is a critical endpoint, as it directly influences disease progression and plays a key role in determining long‐term clinical outcomes for patients with LARC [4, 36, 37, 38]. Patients achieving CR after NACTRT could benefit from less aggressive approaches like wait‐and‐watch strategy or sphincter‐preserving procedures which have a direct impact on the quality of life of rectal cancer patients. Accurate prediction of treatment response is of critical clinical importance, particularly in the context of optimizing therapeutic decision‐making and risk stratification. Several clinicopathological and treatment‐related factors have emerged as independent predictors of pCR. Notably, lower histological tumor grade, as well as lower primary tumor (T) stage and nodal (N) stage, have consistently been associated with a higher probability of achieving pCR. These factors likely reflect a lower baseline tumor burden and more favorable tumor biology, thereby contributing to enhanced treatment sensitivity and improved pathological outcomes [39].

In this study, we modeled the selected radiomic features using five ML classifiers to predict the clinical endpoints of interest and demonstrated that the five selected radiomic features (Table 2) modeled by the RFC performed the best in differentiating complete responders from partial responders following NACTRT, with an AUC: 0.79 ± 0.15, accuracy: 0.72 ± 0.12, *F*1‐score: 0.81 ± 0.07, recall: 0.87 ± 0.11, and precision: 0.77 ± 0.10 for the test cohort. This study also showed the RFC classifier to be the best and most stable performer when compared to SVM, ABC, GBC, or NBC in classifying TR to NACTRT (Table 3, Figure 3A–E). The predictive performance observed through internal cross‐validation in the present study aligns closely with the findings of two large retrospective studies—one conducted by Shen et al. (*n* = 169) and the other by Delli Pizzi et al. (*n* = 72). Both studies explored the predictive value of radiomic features modeled using various ML classifiers, further corroborating the robustness of our results as modeled by the ML classifiers [40, 41]. In the study by Shen et al., radiomic features were extracted from pretreatment PET/CT datasets and subsequently modeled using a RFC. In contrast, the study by Delli Pizzi et al. utilized radiomic features derived from T2‐weighted and diffusion‐weighted MRI sequences, which were then analyzed using partial least squares regression. This study showed good predictive performance in classifying pCR following NACTRT with an AUC of 0.700 using radiomic features alone. However, the predictive performance further improves when radiomic features are combined with MRI‐based clinical features—incorporating factors such as tumor location, tumor volume, distance from internal anal sphincter, extramural vascular invasion, depth of invasion and mesorectal fascia invasion resulting in a higher accuracy, with an AUC of 0.793 as shown in a study by Bulens et al. [42]. In our study, the AUC of the ML classifiers developed using the five selected radiomic features ranged between 0.62 and 0.79, reflecting moderate to good discriminative performance in predicting TR. Notably, the AUC achieved by the RFC was comparable to that reported in prior studies that incorporated both radiomic features and MRI‐derived clinical variables for model development. These observations suggest that the chosen radiomic features alone may encapsulate a significant amount of predictive information, potentially rivaling the performance of more intricate multimodal models (Table 3). The larger study by Shen et al. involving 169 patients of LARC demonstrated a sensitivity, specificity, positive predictive value (PPV), negative predictive value, and accuracy of 81.8%, 97.3%, 81.8%, 97.3%, and 95.3%, respectively in this cohort of patients, with an AUC of 0.94. In comparison to the aforementioned study, the RFC applied to our cohort demonstrated robust predictive performance, achieving an accuracy of 0.72, precision of 0.77, recall of 0.87, *F*1‐score of 0.81, and an AUC of 0.79 for predicting TR to NACTRT. The predictive performance reported by Shen et al. in the Chinese cohort was notably higher than that observed in our study, which may be attributed to the larger training dataset available in their study (*n* = 169) compared to the relatively smaller sample size in our cohort (*n* = 100), potentially providing the classifiers with greater variability and statistical power for feature learning. Another important factor could be the difference in validation methods: our study used 10‐fold cross‐validation, whereas the Chinese study used a train‐test approach. Moreover, the study by Shen et al. implemented ensemble decision trees with optimal splitting criteria, an approach that likely contributed to the improved sensitivity in detecting pCR when assessed using a calibrated threshold. The predictive performance observed during internal cross‐validation suggests that the proposed model may have meaningful clinical utility. However, given the inherent limitations of internal validation, including optimism bias and reduced generalizability, these results should be interpreted with caution. Comprehensive external validation in independent, preferably multicenter cohorts is essential to evaluate the model's reproducibility, generalizability, and performance across heterogeneous patient populations and imaging settings before its adoption in clinical practice.

A large retrospective study by Shin et al. from South Korea involving nearly 900 patients with LARC evaluated radiomic models based on T2‐weighted MRI and diffusion‐weighted MRI (DWI) for predicting pCR after NACTRT in LARC patients and compared their performance with visual assessment by radiologists [43]. The radiomic model was developed on T2W MRI images and diffusion‐weighted MRI images (DWI). This study demonstrated that the model derived from radiomic features extracted from multiparametric MRI outperformed radiologist assessment in predicting pCR. Moreover, the AUCs of the radiomic signatures from T2W, DWI, and merged models were 0.82, 0.79, and 0.82 respectively, between the T2‐weighted and merged models (*p* = 0.49) [43]. The above study though methodologically different from our study, demonstrated the robustness of radiomic signatures in predicting TR using AUC as the performance metrics in contrast to the present study which used several performance metrics derived from radiomic signature modeled by five different validated ML classifiers. Although the study methodology was different, the predictive performance in classifying TR from the radiomic signatures obtained from T2W MRI and modeled by the best ML algorithm in the present study (AUC: 0.79) was comparable to the study by Shin et al. with an AUC of 0.82. Another externally validated study evaluated the predictive value of radiomic and semantic features derived from T2‐weighted (T2W) and apparent diffusion coefficient (ADC) MRI sequences for assessing TR to NACTRT [42]. In this study, the radiomic model alone achieved an AUC of 0.83 (95% CI: 0.73–0.93) for predicting near‐CR to NACTRT, with a sensitivity of 50%, specificity of 90%, and a PPV of 67%. When radiomic features were combined with semantic features from pretreatment T2‐weighted and ADC MRI, the model's performance improved, achieving an AUC of 0.86 (95% CI: 0.75–0.98), with a sensitivity of 52%, specificity of 98%, and PPV of 92%. A second model incorporating both semantic and radiomic features from post‐NACTRT MRI and ADC maps predicted TR with an AUC of 0.84 (95% CI: 0.75–0.94), achieving a sensitivity of 63%, specificity of 88%, and PPV of 67%. The external validation of these models in an independent patient cohort enhanced their robustness and reduced the selection bias in predicting response to NACTRT. The findings of this externally validated radiomic analysis are consistent with the majority of previously reported retrospective studies, which employed nested internal cross‐validation for model development and validation [40, 41, 42, 43, 44, 45, 46, 47].

The RFC demonstrated good discriminative performance, achieving an AUC of 0.79 under a 10‐fold cross‐validation framework. This level of performance is consistent with recently published studies investigating ML‐based prediction of treatment response in rectal cancer. Wen et al. developed a combined clinical–radiomic nomogram that achieved AUCs of 0.86 in the training cohort and 0.83 in the independent validation cohort [33]. Although the AUC observed in the present study is modestly lower, the comparable magnitude of discrimination supports the potential validity of the proposed approach, particularly considering differences in model architecture, feature composition, sample size, and validation strategy. While, Cantürk et al. reported an AUC of 0.837 using a model based on combined radiomic features, demonstrating strong discriminative performance for treatment response prediction [34]. In comparison, the present study extends prior work by systematically evaluating multiple ML algorithms rather than relying on a single classifier, thereby enabling a more comprehensive assessment of model performance and robustness.

Furthermore, radiomic feature extraction was performed using standardized TexRAD software, which enhances methodological reproducibility and reduces variability related to feature computation. The implementation of a structured 10‐fold cross‐validation framework also strengthens the internal validation process by mitigating overfitting and providing more reliable performance estimates. Collectively, these methodological considerations address several limitations reported in the existing literature, including restricted classifier comparisons, non‐standardized radiomic workflows, and insufficient validation strategies.

This study has several limitations. As a retrospective, single‐institution analysis with a relatively small imaging dataset (*N* = 100), external validation of the findings was not feasible. To mitigate the risk of overfitting, internal stratified cross‐validation was employed, however, the limited sample size restricts the generalizability and broader applicability of the results. Since the imaging was done within the institution, the imaging protocols were standardized and uniform for the entire cohort of patients analyzed. Moreover, we extracted radiomic features from both preoperative and posttreatment MRI images to build the model for predicting the response. We were able to achieve relatively good accuracy in developing a robust ML model from the selected features extracted from the MRI dataset, suggesting that this would have clinical implications if validated in a larger cohort of patients in real‐world clinical practice. We do acknowledge that the current strategy of using internal cross‐validation has the limitation of inflating the performance metrics. However, with a limited sample size, cross‐validation is a pragmatic strategy to utilize for developing the predictive models for the clinical endpoint. We only focused on extracting the radiomic features from T2W MRI images, however developing more robust models on radiomic features extracted from multiparametric MRI sequences like DWI and ADC images may have better utility as a predictive imaging biomarker, given its established role as a functional imaging modality for evaluating treatment response in rectal cancer—particularly in patients undergoing NACTRT, diffusion‐weighted imaging (DWI) represents a valuable component of multiparametric MRI protocols. However, at the time of data acquisition for the present study, DWI was not part of the standard institutional MRI protocol. This limitation precluded the inclusion of diffusion—derived quantitative parameters and DWI‐based radiomic features in the current analysis. Recognizing the growing evidence supporting the incremental value of DWI in response assessment, our imaging protocol has since been updated to include standardized DWI acquisition. Future radiomics investigations at our institution are therefore planned to incorporate multiparametric MRI—derived radiomic features, including those extracted from DWI and ADC maps, with the objective of improving predictive modeling for TR following NACTRT. This expanded approach is anticipated to enhance characterization of tumor heterogeneity and treatment‐induced tissue microstructure alterations, thereby potentially improving model performance and clinical applicability.

Despite inherent limitations, the findings of this study hold potential clinical significance for guiding personalized therapy in LARC patients considered for organ‐preserving strategies, utilizing noninvasive radiomic signatures modeled through validated ML algorithms. The developed model is intended for subsequent evaluation in an independent validation cohort, followed by testing on a larger imaging dataset to further assess its generalizability and clinical applicability.

## 5 Conclusion

This study demonstrates that MRI‐derived radiomic features can effectively predict treatment response in LARC patients undergoing NACTRT, with the RFC showing the highest accuracy and stability. These results highlight the potential of combining MRI‐based radiomics with robust ML methods for individualized response assessment.

The proposed predictive model could serve as a noninvasive tool to help identify LARC patients who might be suitable for organ‐preserving treatment strategies. However, its clinical utility and generalizability need to be confirmed through studies with larger cohorts and external validation across multiple centers before it can be applied in routine practice.

## Author Contributions

**Shivakumar Gudi:** writing – original draft, investigation, data curation, supervision, formal analysis, visualization. **Reena Engineer:** conceptualization, data curation, supervision, project administration, visualization, validation, investigation, writing – review and editing. **Ashish Kumar Jha:** methodology, formal analysis, data curation, software, writing – original draft, validation. **Rahul Krishnartry:** methodology, data curation, supervision, project administration, validation. **Jai Prakash Agarwal:** writing – review and editing, supervision, validation, project administration. **Abhinav Puppalwar:** writing – original draft, methodology, data curation, project administration. **Jayant S. Goda:** conceptualization, writing – review and editing, formal analysis, project administration, resources, validation, methodology. **Prashant Nayak:** methodology, software, data curation, investigation, writing – original draft, validation, project administration, visualization.

## Funding

The authors have nothing to report.

## Ethics Statement

The study was approved by the Tata Memorial Hospital Institutional Ethics Committee (IEC no: 900615).

## Consent

Waiver of consent was taken as it was a retrospective study.

## Conflicts of Interest

The authors declare no conflicts of interest.

## Supporting information


**Table S1:** The MR image acquisition parameters.
**Table S2:** The radiomic features extracted from T2W MR images.
**Table S3:** The details of the prediction metrices used for diagnostic accuracy of the machine learning algorithms.

## Data Availability

The data that support the findings of this study are available on request from the corresponding author. The data are not publicly available due to privacy or ethical restrictions.
