# Supplementary material for: MRI Based Radiomics as an Imaging Biomarker for Locally Advanced Carcinoma Rectum: Predicting Tumor Response Following Neoadjuvant Chemoradiotherapy as an Organ Preservation Strategy
Source: Cancer Rep (Hoboken). 2026 May 19;9(5):e70574. doi: 10.1002/cnr2.70574 (PMC13580817; doi:10.1002/cnr2.70574)
Supplement: Supplementary file 1 [file CNR2-9-e70574-s001.docx]

**Supplementary Table-1: Shows the MR image acquisition Parameters**

| **MR Parameters** | **values** |
| --- | --- |
| TR | 3000-6000 |
| TE | 10-30 |
| Image Matrix | 320 X 224 |
| Image thickness | 5mm with interception gap of 1mm |
| Sequence | Axial fast spine echo- T2 weighted |
| Time delay in mage acquisition | 2-3 mins |

TR: RepetitionTime;TE; Echo Time

**Supplementary Table-2: Shows the radiomic features extracted from T2W MR images**

| **1st Order Texture Feature** | **Formulae** | **Remarks** |
| --- | --- | --- |
| Mean | $Mean=\frac{1}{N_{p}}\sum_{i=1}^{N_{p}} X\left( i \right)$ | Mean represents the average gray level intensity within the ROI |
| Standard deviation | $SD=\sqrt{\frac{1}{N_{p}}\sum_{i=1}^{N_{p}} (X\left( i \right)-\overline{X})^{2}}$ | SD represents variation from mean gray-level value SD is small if image is homogenous |
| Skewness | $skewness=\frac{\mu_{3}}{{\sigma^{3}}}=\frac{\frac{1}{N_{p}}\sum_{i=1}^{N_{p}} (X\left( i \right)-\overline{X})^{3}}{\left( \sqrt{\frac{1}{N_{p}}\sum_{i=1}^{N_{p}} (X\left( i \right)-\overline{X})^{2}} \right)^{3}}$ | Skewness is the symmetry of intensity values in an image( ROI). It is zero if the histogram is symmetrical |
| Entropy | $Entropy= \sum_{i=1}^{N_{p}} p\left( i \right){log}_{2}\left( p\left( i \right)+\epsilon\right)$ | Entropy represents irregularity or randomness of intensity value distribution in the ROI.  Here, ϵ is an arbitrarily small positive number (≈2.2×10−16). |
| Kurtosis | $Kurtosis=\frac{\mu_{4}}{{\sigma^{4}}}=\frac{\frac{1}{N_{p}}\sum_{i=1}^{N_{p}} (X\left( i \right)-\overline{X})^{4}}{\left( \frac{1}{N_{p}}\sum_{i=1}^{N_{p}} (X\left( i \right)-\overline{X})^{2} \right)^{4}}$ | Kurtosis is an indication of histogram flatness |
| **GLCM Feature** | **Formulae** | **Remarks** |
| Autocorrelation |  | Autocorrelation is a measure of the magnitude of the fineness and coarseness of texture. |
| Cluster prominence |  | Measure of the skewness and asymmetry of the GLCM. A higher- values implies more asymmetry about the mean while a lower value indicates a peak near the mean value and less variation about the mean. |
| Cluster shade |  | Measure of the skewness and uniformity of the GLCM. A higher cluster shade implies greater asymmetry about the mean |
| Cluster tendency |  | Measure of groupings of voxels with similar gray-level values |
| Contrast |  | Represents the amount of local gray level variation in an image.  A high value of contrast may indicate the presence of edges, noise, or wrinkled textures in the image |
| Correlation |  | Measures the linear dependency of gray levels on those of neighboring pixels.  It provides a measure similar to auto correlation methods |
| Dissimilarity |  |  |
| Homogeneity |  | Measures the smoothness (homogeneity) of the gray level distribution of the image; it is (approximately) inversely correlated with contrast—if contrast is small, usually homogeneity is large |
| Joint average |  | the mean gray level intensity of the i distribution |
| Joint energy |  | Measure of homogeneous patterns in the image. A greater Energy implies that there are more instances of intensity value pairs in the image that neighbour each other at higher frequencies. |
| Joint entropy |  | Measure of the randomness/variability in neighbourhood intensity values |
| IDM |  | Measure of the local homogeneity of an image. IDM weights are the inverse of the Contrast weights (decreasing exponentially from the diagonal i=j in the GLCM). |
| Difference entropy |  | Measure of the randomness/variability in neighbourhood intensity value differences or measures the disorder related to the gray level difference distribution of the image |
| Difference variance |  | Measures the dispersion (with regard to the mean) of the gray level difference distribution of the image or Measure of the randomness/variability in neighbourhood intensity value differences. |
| IDMN |  | Measure of the local homogeneity of an image. IDMN weights are the inverse of the Contrast weights (decreasing exponentially from the diagonal i=j in the GLCM |
| IDN |  | Measure of the local homogeneity of an image. IDN normalizes the difference between the neighboring intensity values by dividing over the total number of discrete intensity values |
| Inverse variance |  | k=0 is skipped, as this would result in a division by 0. |
| Sum entropy |  | Sum of neighbourhood intensity value differences  It measures the disorder related to the gray level sum distribution of the image |
| Joint maximum probability |  | Represents the occurrences of the most predominant pair of neighboring intensity values. |
| **Shape Feature** | **Formulae** | **Remarks** |
| Elongation | $Elongation=\sqrt{\frac{\lambda_{minor}}{\lambda_{major}}}$ | Elongation shows the relationship between the two largest principal components in the ROI shape  λmajor and λminorare the lengths of the largest and second largest principal component axes. The values range between 1 (where the cross section through the first and second largest principal moments is circle-like (non-elongated)) and 0 (where the object is a maximally elongated. |
| Area | $A_{\mathrm{pixel}}= \sum_{k=1}^{N_{v}} A_{k}$ | The surface area of the ROI A pixel is approximated by multiplying the number of pixels in the ROI by the surface area of a single pixel Ak |
| Sphericity | $Sphericity=\frac{2\sqrt{\pi A}}{P}$  Where  $A=\pi r^{2}$ | Sphericity is the ratio of the perimeter of the tumor region to the perimeter of a circle with the same surface area as the tumor region and therefore a measure of the roundness of the shape of the tumor region relative to a circle. |
| Short axis | $Short axis=4\sqrt{\lambda_{minor}}$ | This feature yields the second largest axis length of the ROI-enclosing ellipsoid and is calculated using the largest principal component λminor |
| Long Axis | $Long axis=4\sqrt{\lambda_{major}}$ | This feature yields the largest axis length of the ROI-enclosing ellipsoid and is calculated using the largest principal component λmajor |
| Perimeter | $P_{i}=\sqrt{{{(a}_{i}-b_{i})}^{2}}$  Where  $P= \sum_{i=1}^{N_{f}} P_{i}$  ai and bi are vertices of the ith line in the perimeter mesh. Total perimeter is then obtained by taking the sum of all calculated sub-areas |  |

GLCM : **Grey level co-occurrence matrix**; MPP: **Mean of Positive Pixel** ; IDM: Inverse Difference Moment; IDMN: Inverse Difference Moment Normalized; IDN: Inverse Difference Normalized

**Supplementary Table-3: Shows the details of the prediction metrices used for diagnostic accuracy of the Machine learning algorithms.**

| **Diagnostic Metrics** | **Formulae** |
| --- | --- |
| Accuracy | ((TP+TN) / Population) |
| Precision | (TP / (TP+FP)) =PPV |
| Recall | (TP / (TP+FN)) |
| F1 | (2 * ((Precision*Recall)/(Precision + Recall)) |
